# Sputum from Individuals with Primary Ciliary Dyskinesia Drives M2-like Macrophage Polarization

**DOI:** 10.1007/s00408-025-00868-6

**Published:** 2026-01-26

**Authors:** Jenny Wåhlander, Tobias Schmidt, Christine R. Hansen, Robin Kahn, Lisa I. Påhlman

**Affiliations:** 1 Division of Infection Medicine, Department of Clinical Sciences Lund, Lund University, Lund, Sweden; 2 Wallenberg Centre of Molecular Medicine, Lund University, Lund, Sweden; 3 Department of Pediatrics, Skåne University Hospital Lund, Lund, Sweden; 4 Division of Rheumatology, Department of Clinical Sciences Lund, Lund University, Lund, Sweden; 5 Division of Pediatrics, Department of Clinical Sciences Lund, Lund University, Lund, Sweden; 6 Department of Infectious Diseases, Skåne University Hospital Lund, Lund, Sweden

**Keywords:** Primary ciliary dyskinesia, Macrophages, Inflammation, Airways

## Abstract

**Introduction:**

Primary ciliary dyskinesia (PCD) is a rare, congenital condition in which impaired ciliary function leads to bronchiectasis and progressive lung function decline. Bronchiectasis development is believed to involve infection and inflammation but is incompletely understood. Macrophages play a central role in cellular immune response, contributing to both pathogen clearance and immunoregulation. Depending on local stimuli, macrophages are polarized towards pro-inflammatory (M1) or pro-resolution/phagocytic (M2) phenotypes. This study aims to investigate the effects of PCD sputum on macrophage polarization.

**Methods:**

Sputum from 27 individuals with PCD and seven healthy controls were used to stimulate healthy monocyte-derived macrophages. Macrophage polarization was determined by surface markers, phagocytic ability and cytokine production using flow cytometry and immunoassays.

**Results:**

Macrophages stimulated with PCD sputum exhibited enhanced phagocytosis (MFI 194268 vs. 58235, *p* = 0.0002), increased expression of M2-associated surface markers CD163, CD206 and CD16, and reduced secretion of proinflammatory cytokines IL-6 (10.38 vs. 113.22 pg/ml, *p* = 0.0013) and IL-1β (0.75 vs. 3.60 pg/ml, *p* < 0.0001). Concurrently, expressions of M1-associated surface markers CD40 and CD80 were reduced.

**Conclusion:**

PCD sputum induced a phagocytosis prone, M2-like phenotype in healthy macrophages.

**Supplementary Information:**

The online version contains supplementary material available at 10.1007/s00408-025-00868-6.

## Introduction

Primary ciliary dyskinesia (PCD) is a rare congenital disorder characterized by dysfunctional or absent cilia, leading to impaired mucociliary clearance and recurrent airway infections [1, 2]. Gradually, PCD patients develop bronchiectasis, characterized by irreversible dilation of the bronchi and progressive lung function decline. Current treatment regimens include regular airway clearance techniques and saline inhalations to improve mucus mobilization, and prompt start of antibiotics during exacerbations [1, 3]. The pathophysiology behind bronchiectasis in PCD remains poorly understood, but a “vicious vortex” model has been proposed where airway dysfunction, inflammation, infection and structural damage perpetuate one another [4, 5]. Previous research has predominantly focused on neutrophils, as airway inflammation in PCD patients is neutrophil dominated [6–9]. Macrophages represent less than 10% of airway immune cells [9], but despite their lower abundance, macrophages are central to airway homeostasis by initiating and resolving inflammation, recruiting neutrophils to the lung, and clearing pathogens and cellular debris through phagocytosis.

Lung macrophages include alveolar (AMs) and interstitial macrophages (IMs), both originating from self-renewing embryonic progenitors. During infection, circulating monocytes are recruited to the lungs, supplementing or replacing the resident macrophage population [10]. In response to the surrounding environment, macrophages adopt various phenotypes, a process termed polarization. In vitro, macrophages are traditionally polarized into M1 and M2 phenotypes with different functional properties. The M1 phenotype is induced by inflammatory stimuli such as LPS or TNF/IFN-γ. Functionally, M1 macrophages produce increased levels of proinflammatory mediators, including IL-1β and IL-6, facilitate the recruitment of neutrophils and monocytes, promote T-helper1-cells, and enhance pathogen killing at the site of infection. Conversely, M2 macrophages are associated with resolution of inflammation, tissue repair, and enhanced phagocytosis and efferocytosis. M2 also secrete increased levels of IL-10, which suppresses M1 activation. The M2 phenotype can be induced in vitro by glucocorticoids or anti-inflammatory cytokines, including IL-10 and TGF-β [11–14]. Importantly, the M1/M2 model represents a simplification, particularly in vivo where macrophages often exhibit characteristics of both phenotypes. Moreover, the functional properties traditionally ascribed to M1 and M2 macrophages can be observed across the spectrum of polarization states, albeit to varying degrees [12, 15]. In this study, we use “M1-like” to describe macrophages with upregulated expression of M1-associated surface markers or production of proinflammatory cytokines. “M2-like” is used to describe macrophages with increased phagocytic ability, anti-inflammatory cytokine secretion or M2-associated surface markers.

Despite the central role of macrophages in regulating inflammation, their involvement in PCD airway disease is poorly understood. Notably, several studies have reported increased macrophage numbers in bronchiectasis [16, 17] and a recent study demonstrated that mucus plugs in non-cystic fibrosis bronchiectasis are populated by IL-1β-producing macrophages, indicating a central role for macrophages in the pathogenesis of bronchiectasis [18]. Moreover, PCD sputum has been shown to inhibit efferocytosis in M2 macrophages, suggesting an altered macrophage function in PCD airways [9]. Given the knowledge gaps of macrophages’ role in PCD, the objective of the present study was to investigate the impact of the PCD airway environment on macrophage polarization patterns and effector functions.

## Methods

## Study Population

Children and adults with a PCD diagnosis and follow-ups at Skåne University Hospital in Lund, Sweden, were eligible for enrolment in the study. Sample collection was performed between 2022 and 2024, with each study participant contributing with one sputum sample during a follow-up visit at the clinic. Patients unable to expectorate were excluded. Information about sputum culture results and clinical data from the time of sputum sampling were collected from the patients’ hospital records. Exacerbation was defined according to Lucas et al. [19]. Healthy adults were eligible for participation as healthy controls.

### Sputum Collection and Sample Preparation

Induced sputum samples were expectorated with physiotherapist-led chest exercises. Samples were dissolved with 0.1% dithiothreitol (DTT) (Sigma-Aldrich) as described [20]. One aliquot of the dissolved sputum was collected and stored at − 80 °C for later DNA analysis. The remaining sputum sample was centrifuged at 850 rcf for 10 min followed by sterile filtration of the cell-free supernatant through 0.22 μm syringe filters. The supernatant was collected and stored at − 80 °C.

## DNA Extraction and Quantitative PCR

Extraction of sputum DNA and quantification of bacterial DNA was performed as described previously [21].

## Cytokine Concentrations in Sputum

IL-6 in sputum was quantified with ProQuantum Immunoassays (Invitrogen) at a 1:2 dilution. IL-1β was measured with MesoScale U-PLEX immunoassay (MesoScale Diagnostics) in a 1:5 dilution.

## Monocyte Isolation and Macrophage Stimulation

Peripheral blood mononuclear cells (PBMCs) were isolated from heparinized blood drawn from one healthy volunteer as described previously [22]. Monocytes were differentiated into macrophages in 24-well tissue culture plates (500 000 cells/well at 1 × 10^6^cells/ml) by incubation at 37 °C for 5 days in RPMI-1640 with 10% normal human serum (NHS) (Sigma-Aldrich) and 20 ng/ml M-CSF (RnD Systems). Cell medium was changed to RPMI-1640 with 10% NHS and 10% sputum supernatant before an additional two-day incubation at 37 °C. For technical controls, macrophages were incubated in RPMI-1640 with 10% NHS for naïve macrophages (M0), with addition of 10 ng/ml LPS (InvivoGen) + 10 ng/ml IFNγ (RnD Systems) for M1 and 10 nM Dexamethasone (MP Biomedicals) + 25 ng/ml IL-10 (RnD Systems) for M2 [23]. Following the two-day incubation, cells were detached from the tissue culture plates upon a 15-minute incubation with Versen (PBS + 1mM EDTA) in 2 consecutive sets. Cells were prepared at seven separate occasions within a 16-day period. On each occasion, one healthy control and at least one pediatric and one adult PCD sputum sample were processed.

## Surface Marker Detection with Flow Cytometry

Upon detachment, polarized macrophages were counted in a XN-350 differential analyzer (SYSMEX), washed in PBS and then incubated for 30 min in the dark at room temperature with necrosis marker LIVE/DEAD Scarlet and antibodies targeting surface markers associated with M1 macrophages (CD80, CD86, PD-L1, HLA-DR, CD40) and with M2 macrophages (MerTK, CD206, CD163 and CD16) [22–28]. 100 µl FACS buffer (PBS with 0.5% BSA) per 100 000 cells with of 1:200 (v/v) dilution of each antibody and 1:1000 (v/v) necrosis marker LIVE/DEAD Scarlet was used. Fluorophores and manufacturers of all markers are listed in supplementary Table 1. The macrophages were washed in PBS and incubated for an additional 15 min in the dark at room temperature with 1:200 Annexin V APC, as an apoptosis marker. The surface markers were analyzed, measured as Median Fluorescent Intensity (MFI) in a CytoFLEX Flow Cytometer (Beckman Coulter). Following doublet exclusion, at least 5000 live macrophages per sample were analyzed. Viability staining with Annexin V and LIVE/DEAD ensured that live macrophages were analyzed. Gating strategy is presented in supplementary Fig. 1.

### Phagocytosis Assay

Macrophages harvested simultaneously as those used for surface marker analysis were incubated with 20 µl pHrodo™ Green *S. aureus* BioParticles™ Conjugate for Phagocytosis (Invitrogen) per 100 000 macrophages in 100 µl PBS with 10% NHS for 1 h at 37 °C. As negative control the phagocytosis assay was performed with M0 macrophages on ice. Phagocytosis was measured as MFI of pHrodo in the CytoFLEX Flow Cytometer. At least 4000 macrophages per sample were examined after doublet exclusion. Gating strategy is presented in supplementary Fig. 2. Technical controls can be viewed in supplementary Fig. 3A.

## Cytokine Secretion of Polarized Macrophages

250 000 macrophages at 1 × 10^6^cells/ml were stimulated with sputum for two days in 48-well tissue culture plates as described above. After stimulation, cells were washed three times using 500 µl PBS before addition of 300 µl fresh cell medium RPMI with 10% NHS to the tissue culture wells. After incubation at 37 °C for an additional 72 h, the cell medium was collected and stored at − 80 °C. Cell medium concentrations of IL-1β were measured using MesoScale, IL-6 and IL-10 with ProQuantum as described in *“Cytokine concentrations in sputum”*. Technical controls are presented in supplementary Fig. 3B.

### Statistical Analysis

Statistical analysis was performed using GraphPad Prism 10 (GraphPad Software, San Diego, CA) and are presented with median and range unless otherwise specified. Mann-Whitney U-test was used comparing groups. Two-tailed *p* < 0.05 was considered statistically significant. Spearman’s rank correlation coefficient was used to analyze the occurrence of correlation between measurements.

### Ethics Approval and Consent to Participate

The study was approved by the Regional Ethical Review Board of Lund University (2018/54) and the Swedish Ethical Review Authority (2024-00361-01). Written informed consent was obtained from all participants or, in the case of patients younger than 15, their guardians after assent from the patients. All experiments were performed in accordance with the Declaration of Helsinki.

## Results

### Study Population and Sputum Characteristics

**Fig. 1:**
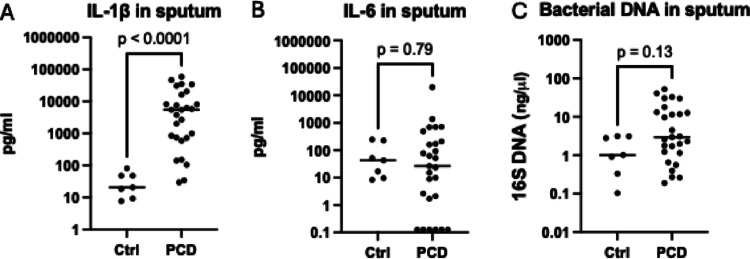
Sputum of PCD patients contain higher levels of IL-1β. Comparison of IL-1β (detection range 0.75 pg/ml − 19000 pg/ml) (**A**), IL-6 (detection range 0.128 pg/ml − 20000 pg/ml) (**B**) and bacterial 16 S DNA (detection range 0.001–1000 ng/µl) (**C**) levels in sputum of PCD patients (*n* = 27) and healthy controls (*n* = 7). Bars represent median values. In six adult samples IL-6 levels were lower than the assay range and therefore set to 0.128 pg/ml, equivalent to the lowest detectable final concentration. One adult sputum sample had IL-6 levels higher than the assay range and therefore set to 20000 pg/ml corresponding to the highest detectable final concentration

Sputum samples were collected from 27 PCD patients, of which ten were children. In addition, seven healthy individuals were recruited as controls. The participants’ baseline characteristics are presented in Table 1. The PCD cohort had a median FEV1pp of 75% and the most common pathogens identified in sputum cultures were Haemophilus influenzae followed by Pseudomonas aeruginosa and Staphylococcus aures. A complete list of pathogens in sputum cultures is presented in supplementary Table 2. Sputum from PCD patients had significantly higher concentrations of IL-1β compared to healthy controls (Fig. 1A), whereas IL-6 and total bacterial DNA were comparable between the groups (Fig. 1B, C). Patients with a positive sputum culture had higher concentrations of bacterial DNA and IL-1β in sputum but lower levels of IL-6 (supplementary Table 3).

**Table 1:** Patient characteristics

Demographic and clinical data	PCD *n* = 27	Controls *n* = 7
Age (years) median (range)	21.9 (7.6–60)	46 (29–62)
Female *n* (%)	14 (52)	5 (71)
FEV1pp median (range)	75 (19–116)	
Genetically confirmed diagnosis *n* (%)	21 (78)	
Clinical diagnosis or unknown *n* (%)	6 (22)	
Ongoing exacerbation *n* (%)	4 (15)	
Positive sputum culture *n* (%) • *Haemophilus influenzae* • *Pseudomonas aeruginosa* • *Staphylococcus aureus*	19 (70) 12 (44) 5 (19) 3 (11)	
Negative sputum culture *n* (%)	6 (22)	
No sputum culture *n* (%)	2 (7)	
Presence of bronchiectasis *n* (%)^a^	22 (81)	
Inhaled corticosteroid treatment *n* (%)	15 (56)	
Azithromycin treatment *n* (%)	6 (22)	
Other antibiotic treatment *n* (%)	5 (19)	

FEV1pp = Forced expiratory volume in one second in percent of predicted. ^a^ Diagnosed by chest CT (*n* = 20) or chest tomosynthesis (*n* = 7)

Demographic and sputum characteristics were further compared between children and adults within the PCD patient cohort. Children had a significantly higher lung function compared to adults, and a lower proportion of children showed radiographic evidence of bronchiectasis on chest x-rays. The prevalence of positive sputum cultures, inhaled cortisone treatment or antibiotic treatment did not vary between the age groups (supplementary Table 4).

### Macrophages Stimulated with PCD Sputum Display an M2-like Surface Marker Pattern

**Fig. 2:**
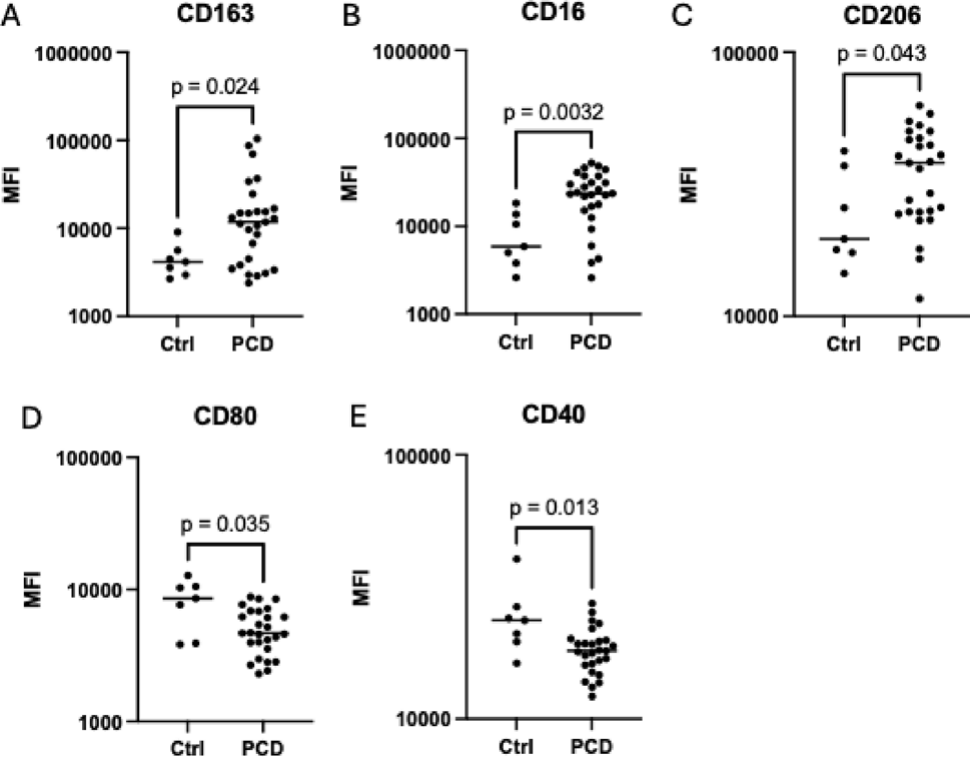
Macrophages stimulated with PCD sputum express M2-associated surface markers. Macrophages were stimulated with sputum from healthy controls (*n* = 7) and PCD patients (*n* = 27), and surface marker expression was analyzed using flow cytometry. The figure shows Median Fluorescent Intensities (MFI) of M2-associated surface markers CD163 (**A**), CD16 (**B**) and CD206 (**C**), and M1-associated surface markers CD80 (**D**) and CD40 (**E**). Bars represent median MFI values

Macrophages exposed to PCD sputum exhibited increased surface expression of M2-associated markers CD163, CD16 and CD206 compared to those stimulated with healthy control sputum (Fig. 2A–C). Conversely, the expression of M1-associated surface markers CD80 and CD40 was reduced in macrophages stimulated with PCD sputum relative to healthy control sputum (Fig. 2D, E). No differences were observed in the expression of M2-related MerTK or M1-associated surface markers CD86, HLA-DR and PD-L1 (supplementary Table 5). Collectively, the data indicate that macrophages stimulated with PCD sputum exhibit a more M2-like surface marker profile than macrophages stimulated with healthy control sputum.

### PCD Sputum Induced M2-like Functions in Macrophages

**Fig. 3:**
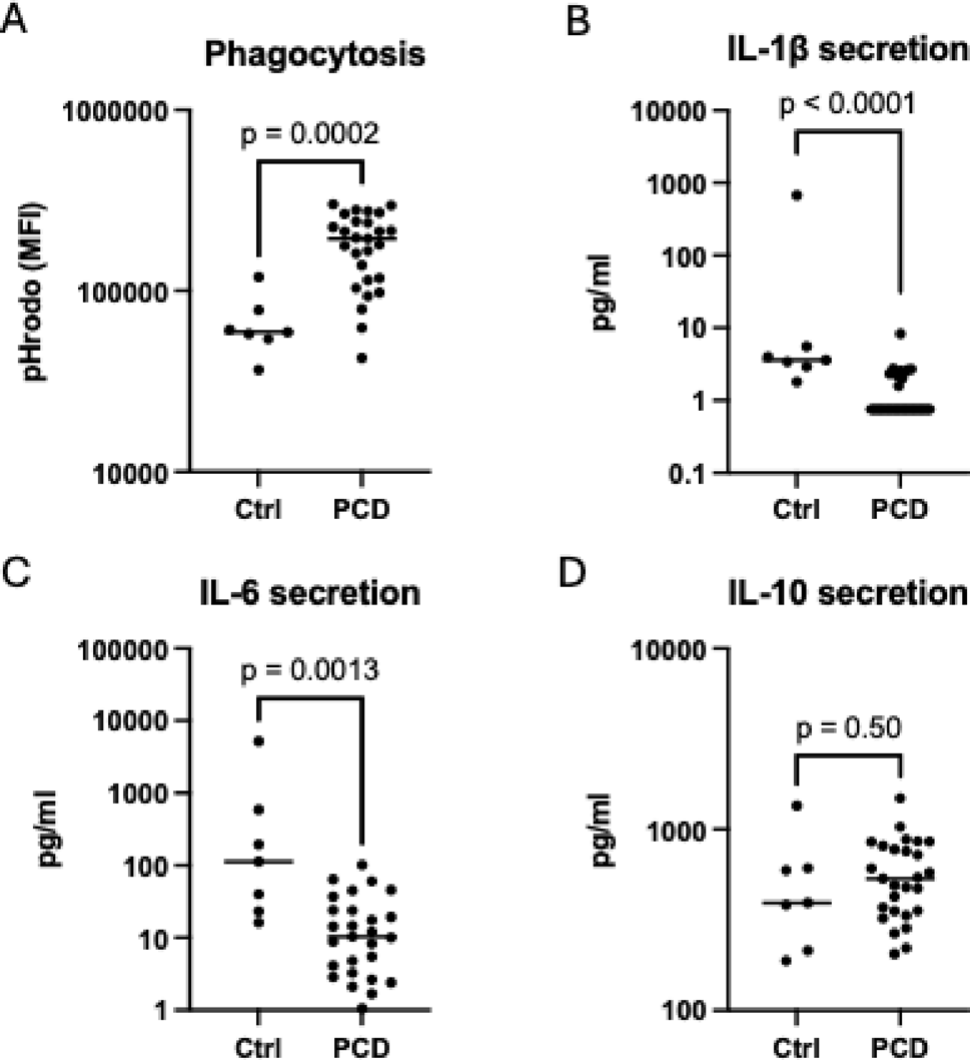
PCD sputum enhances macrophage phagocytosis and reduces IL-1β and IL-6 secretion. Macrophage effector functions were compared in macrophages stimulated with sputum from healthy controls (*n* = 7) or PCD patients (*n* = 27). (**A**) Phagocytosis was assessed by incubating sputum-polarized macrophages with pHrodo Green *S. aureus *BioParticles. Macrophage fluorescence was then quantified using flow cytometry and visualized as Median Fluorescence Intensity (MFI). (**B**–**D**) Sputum-polarized macrophages were washed and incubated in fresh cell medium for 72 h. Levels of IL-1β (detection range 0.75 pg/ml–19000 pg/ml) (**B**), IL-6 (detection range 0.128 pg/ml–20000 pg/ml) (**C**) and IL-10 (detection range 0.128pg/ml–10000 pg/ml) (**D**) in the cell medium were thereafter analyzed using MesoScale or ProQuantum assays. In 13 adult and 4 pediatric samples, IL-1β levels were below detection range and were therefore set to 0.128 pg/ml, equivalent to the lowest detectable final concentration. Bars represent median MFI or cytokine values

Next, functional properties of sputum-induced macrophages were analyzed. Sputum from PCD patients generated macrophages with a greater phagocytic ability than healthy control sputum, matching an M2-like phenotype (Fig. 3A). Concurrently, macrophages stimulated with PCD sputum showed lower secretion of M1-associated cytokines IL-1β and IL-6, congruent with the M2-like phenotype (Fig. 3B, C). However, no difference was observed in secretion of M2-associated IL-10 (Fig. 3D).

**Fig. 4:**
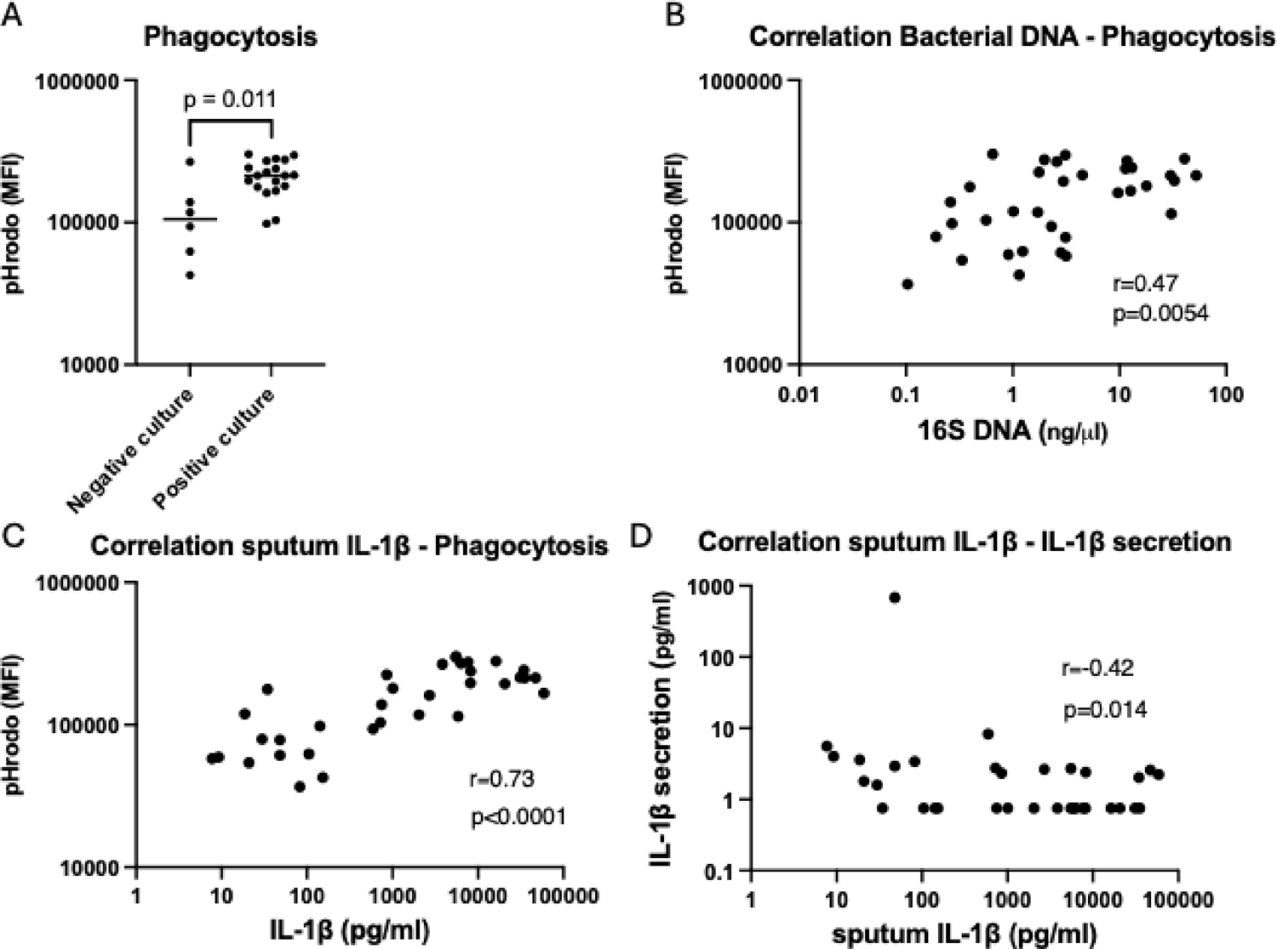
Macrophage effector functions correlate with sputum properties. (**A**) Shows Median Fluorescent Intensity (MFI) of phagocytosis in macrophages stimulated with sputum from PCD patients with either negative (*n* = 6) or positive (*n* = 19) sputum culture. Bars represent median MFI values. (**B**, **C**) Spearman’s rank correlation coefficient was used to assess correlations between phagocytosis and bacterial load and sputum IL-1β in sputum-polarized macrophages (*n* = 34). (**D**) Illustrates Spearman’s rank correlation coefficient of sputum IL-1β and IL-1β secretion of the sputum stimulated macrophages. The correlation between IL-1β sputum concentration and IL-1β secretion remains significant when the outlier, with IL-1β secretion of 677 pg/ml, is excluded (*p* = 0.0278, *r* = − 0.383)

Sputum samples with positive sputum cultures induced higher levels of phagocytosis (Fig. 4A), and the phagocytic ability correlated positively with sputum concentrations of bacterial DNA and IL-1β (Fig. 4B, C). In addition, sputum IL-1β correlated negatively with macrophage secretion of IL-1β (Fig. 4D). Thus, high sputum levels of bacterial DNA and IL-1β are associated with M2-related macrophage functions. Correlation data between sputum content and macrophage functions can be viewed in Supplementary Table 6.

### Effects of Treatment Regimens and Age on Macrophage Polarization

Several PCD patients in the study were treated with immunomodulatory therapies including inhaled corticosteroids and Azithromycin. Sputum from patients receiving these treatments did not differ significantly concerning induction of macrophage cytokine secretion, phagocytosis, or expression of surface markers CD40, CD80, CD163 and CD16. However, patients receiving corticosteroid inhalations had higher expression of CD206, suggesting an M2-like polarization. Nevertheless, treatment regiments did not significantly impact overall macrophage functions (supplementary Table 7).

Both pediatric and adult PCD patients participated in the study, whereas the healthy control group consisted exclusively of adults. When comparing PCD sputum from adults and children, no significant differences were observed in macrophage surface marker expression or phagocytosis. Sputum from adults induced significantly lower IL-1β- and IL-6-secretion, suggesting a more pronounced M2-like polarization (supplementary Table 7). These findings suggest that the inclusion of children in the PCD group did not account for the polarization differences between the PCD cohort and healthy controls.

## Discussion

In this study, we demonstrate that macrophages stimulated with sputum from patients with PCD adopt an M2-like phenotype, with enhanced phagocytic ability and reduced secretion of the pro-inflammatory cytokines IL-1β and IL-6. However, compared to sputum from healthy controls, PCD sputum contained elevated IL-1β levels, a pro-inflammatory cytokine predominantly produced by M1 macrophages [11, 12, 14] that is linked to disease severity and neutrophilic inflammation in patients with bronchiectasis [29]. Similar M2-like responses have been observed in other inflammatory and infectious conditions, including monocytes with increased efferocytic ability and resistance to cytokine production upon exposure to synovial fluid from patients with juvenile idiopathic arthritis [30], as well as endotoxin tolerance in monocytes from individuals with sepsis and cystic fibrosis [31]. These observations raise the possibility that the macrophage response to PCD sputum may not be PCD-specific, but rather a generalized response to infection and inflammatory factors in the airways. Comparative studies involving other lung conditions would be of value in future investigations. Nevertheless, whether the M2-like phenotype is beneficial or not in PCD airway disease remains unclear. While M2-like macrophages may help counteract an excessive inflammatory response, their anti-inflammatory effects could also impair microbial defense, potentially perpetuating inflammation. Notably, Blanter et al reported reduced efferocytosis in PCD sputum-stimulated M2-polarized macrophages [9]. This contrasts our finding that PCD sputum enhances phagocytosis, a clearance mechanism functionally related to efferocytosis. Though it is possible that PCD sputum enhances phagocytosis while impairing efferocytosis, the methodologies and research questions of the two studies differ: Blanter et al assessed the effects of a 3-hour incubation with PCD sputum on M2 macrophages compared to unstimulated M2 macrophages, rather than comparing M0 macrophages stimulated with PCD- or healthy control-sputum. Hence, whether reduced efferocytosis is specific to PCD sputum or a general effect of sputum exposure requires further investigation.

Glucocorticoids and Azithromycin are commonly used therapies in PCD patients, as well as potential inducers of M2-like phenotypes in macrophages [11–13, 32]. For example, Azithromycin reduces pulmonary exacerbations in PCD patients [33] and improves alveolar macrophage efferocytosis in vitro [34]. Neither inhaled corticosteroids nor immunomodulating Azithromycin were associated with more prominent M2-traits in our study, although the statistical powers of our analyses are weak due to the relatively small sample sizes. Positive sputum cultures and elevated levels of bacterial DNA were associated with increased phagocytic activity, suggesting a link to infection. However, numerous factors could confound these results, including inflammatory cytokines, microbiome composition, and other immune cells. Nevertheless, this study was not designed to identify specific factors in sputum driving macrophage polarization. Consequently, the factor(s) responsible for the M2-like polarization in response to PCD sputum remain unidentified. 

This study has several limitations. The single-center design and relatively small sample size may restrict the generalizability of the findings. Another important limitation is the use of blood monocyte-derived macrophages instead of airway macrophages that are challenging to obtain. Although alveolar macrophages would be more representative of the airways, a portion of the lung macrophage pool derives from circulating monocytes, particularly during infection when recruitment from the circulation increases [10]. Additionally, all experiments were conducted using macrophages from the same healthy donor. While this was deliberate to standardize responses across sputum samples, it also reduces generalizability to other individuals’ macrophages. Moreover, using healthy donor macrophages rather than PCD-derived macrophages may have influenced the results. Although chemotactic responses appear similar between PCD and healthy macrophages [35], PCD monocytes secrete higher levels of inflammatory cytokines in response to LPS compared to healthy controls [36]. Furthermore, our findings reflect in vitro macrophage responses to the PCD sputum environment, rather than in vivo polarization patterns within the PCD airways. Various factors and compensatory mechanisms may influence inflammation and macrophage polarization in the lung, where macrophages likely exist along a continuum of M1/M2 polarization [12, 15]. Supporting this, macrophages stimulated with PCD sputum did not exhibit IL-10 secretion or MerTK upregulation, nor reduced expression of M1 markers PD-L1, HLA-DR and CD86, as seen in classical M2 phenotypes. While these results do not contradict M2-like macrophage polarization, they demonstrate the complexity of macrophage phenotypes. Moreover, macrophage polarization may vary across airway compartments. For instance, in chronic obstructive pulmonary disease (COPD), a condition sharing features of chronic airway infection and inflammation with PCD, an M1 phenotype predominately localizes to the alveolar space, while M2 macrophages reside in the luminal compartment [37]. As sputum likely reflects the luminal environment, our findings with sputum-stimulated macrophages align with the observed M2-like polarization in COPD.

## Conclusions

This study provides novel insights into macrophage polarization in response to the PCD airway environment, revealing an M2-like phenotype characterized by increased phagocytosis, reduced secretion of pro-inflammatory cytokines and surface marker patterns associated with M2 macrophages. Future investigations should explore the underlying mechanisms driving this polarization and its implications for PCD pathophysiology.

## Supplementary Information

Below is the link to the electronic supplementary material.

Supplementary Material 1

## Data Availability

All data and information of the materials used can be obtained by contacting the corresponding author.
