# Supplementary material for: Sputum from Individuals with Primary Ciliary Dyskinesia Drives M2-like Macrophage Polarization
Source: Lung. 2026 Jan 26;204(1):6. doi: 10.1007/s00408-025-00868-6 (PMC12832589; doi:10.1007/s00408-025-00868-6)
Supplement: Supplementary file 1 — Supplementary Material 1 [file 408_2025_868_MOESM1_ESM.pdf]

**Supplementary table 1. Flow cytometry markers**

| Marker | Fluorophore | Function | Clone | Manufacturer |
|---|---|---|---|---|
| CD80 | Alexa Fluor® 488 | M1 | 2D10 | Biolegend |
| HLA-DR | PE-Cy7 | M1 | G46-6/L243 | BD Biosciences |
| CD40 | BV510 | M1 | 5C3 | BD Biosciences |
| PDL1 | BV421 | M1 | MIH1 | BD Biosciences |
| CD86 | BV650 | M1 | FUN-1 | BD Biosciences |
| MerTK | PE | M2 | 590H11G1E3 | Biolegend |
| CD16 | PerCP/Cyanine5.5 | M2 | 3G8 | Biolegend |
| CD163 | PE-CF594 | M2 | GHI/61 | BD Biosciences |
| CD206 | APC/Fire™ 750 | M2 | 15-2 | Biolegend |
| LIVE/DEAD | Scarlet | Necrosis | | Invitrogen |
| Annexin V | APC | Apoptosis | | BD Biosciences |

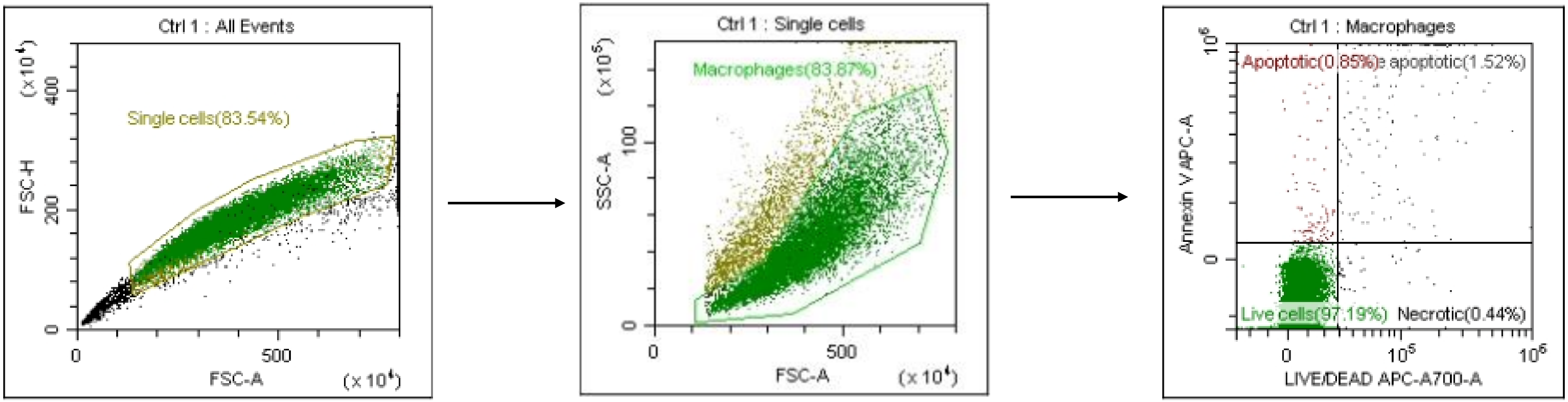


**Supplementary figure 1. Gating strategy surface markers**

Healthy control 1 used as an example. Duplet exclusion was attained by FSC-H/FSC-A, gating Single cells. Macrophages were gated by analyzing Single cells by SSC-A/FSC-A. Apoptotic, late apoptotic and necrotic cells within the Macrophages population were excluded by gating away cells with higher levels of Annexin V and/or LIVE/DEAD. The remaining macrophages were deemed Live cells. Surface markers were measured in MFI (Median Fluorescent Intensity) in the Live cells group.

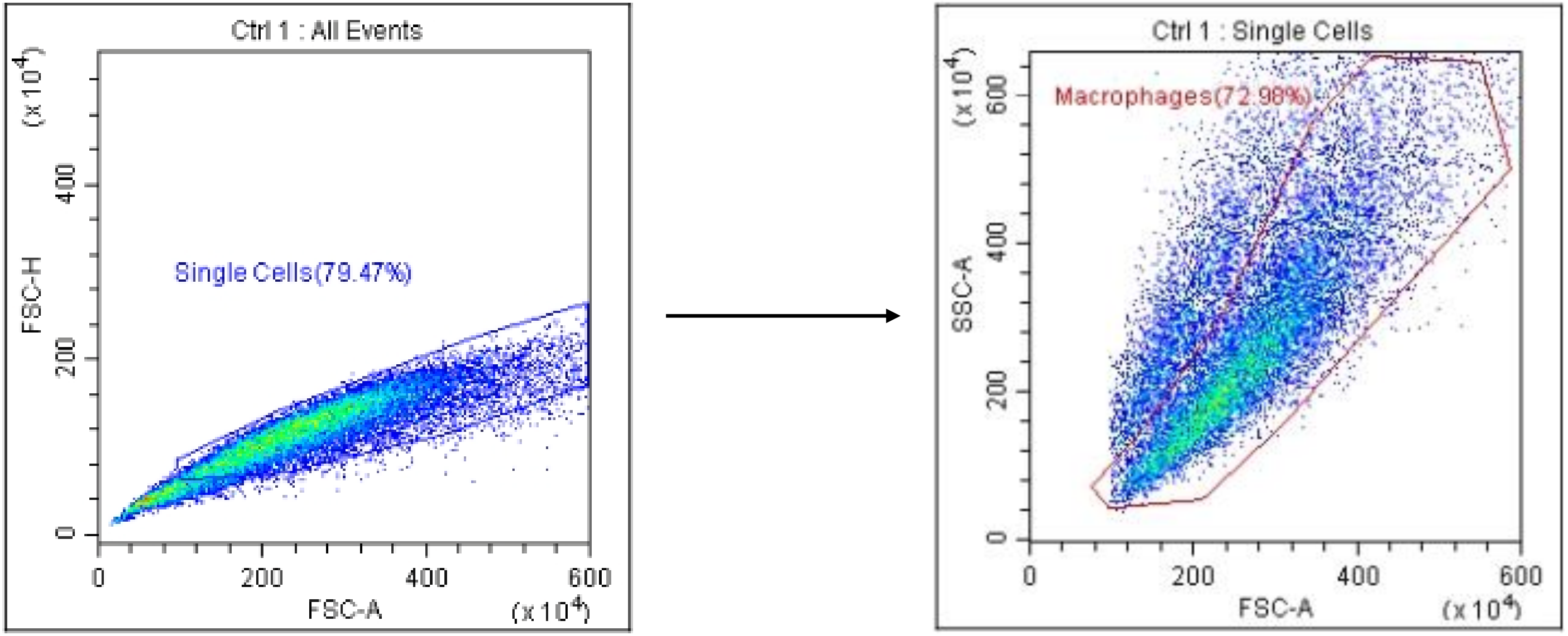


**Supplementary figure 2. Gating strategy phagocytosis**
Healthy control 1 used as an example. Duplet exclusion was attained by FSC-H/FSC-A, gating Single cells. Macrophages were gated by analyzing Single cells by SSC-A/FSC-A. MFI (Median Fluorescent Intensity) of pHrodo was measured in the Macrophages group.

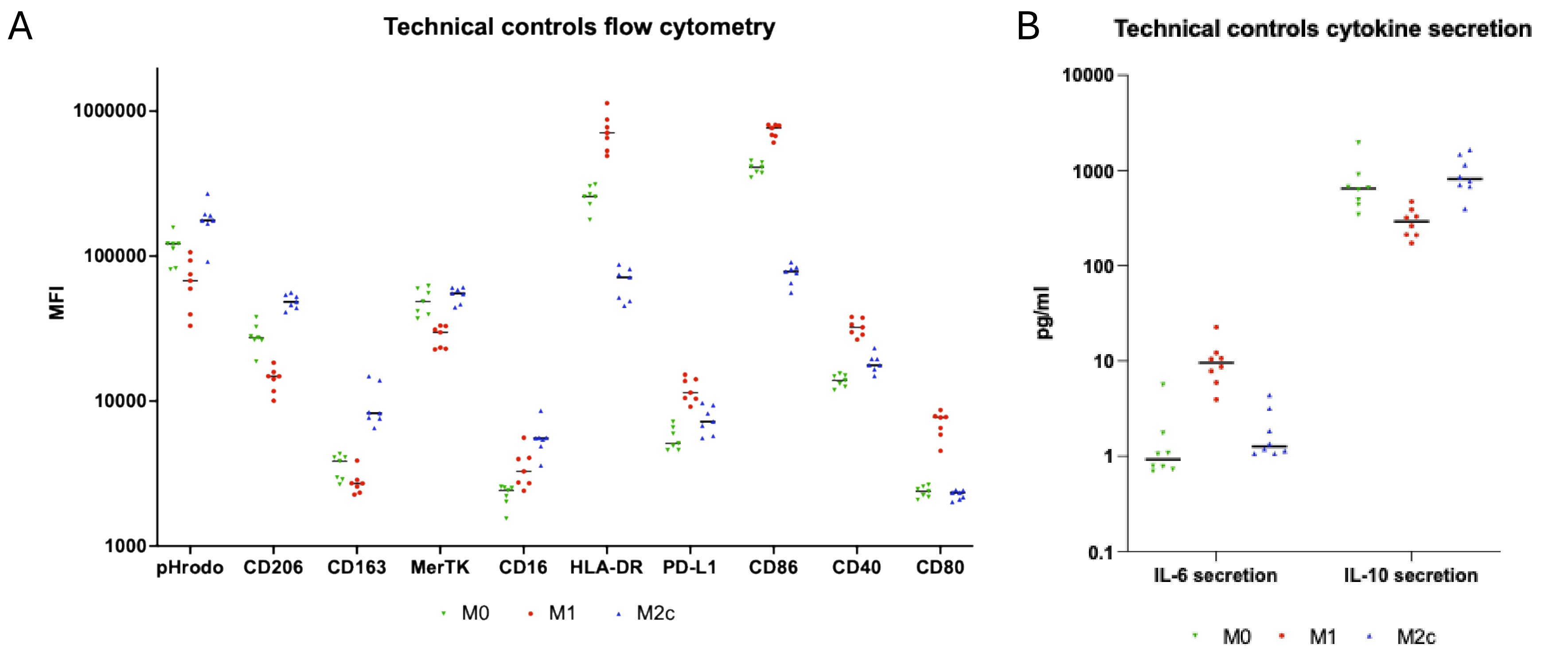


**Supplementary figure 3. Technical controls**

Seven separate experiments were performed to stimulate macrophages with sputum samples from PCD patients and healthy controls. The figure shows technical controls M0, M1 and M2c from all seven occasions. (A) Flow cytometry analysis of surface marker expression after polarization. (B) Measurements of cytokine secretion from polarized macrophages.

| **Supplementary table 2. Pathogens in regular sputum culture** | PCD | |
|---|---|---|
| | Children (n=10) | Adults (n=17) |
| *Haemophilus influenzae* n | 4 | 8 |
| *Pseudomonas aeruginosa* n | 0 | 5 |
| *Staphylococcus aureus* n | 2 | 1 |
| *Moraxella catarrhalis* n | 1 | 2 |
| *Klebsiella pneumoniae* n | 1 | 0 |
| *Streptococcus equi* n | 1 | 0 |
| *Klebsiella oxytoca group* n | 0 | 1 |
| *Enterobacter cloacae complex* n | 0 | 1 |
| *Aspergillus species* n | 1 | 0 |
| *Aspergillus niger complex* n | 0 | 1 |
| *Aspergillus fumigatus* n | 0 | 2 |
| *Exophiala dermatitidis* n | 0 | 1 |
| No relevant pathogens n | 4 | 2 |
| No culture n | 0 | 2 |

| **Supplementary table 3. Sputum characteristics comparing sputum samples with positive and negative sputum culture** | Positive sputum culture | | Negative sputum culture | | | Spearman's correlation to 16S DNA | |
|---|---|---|---|---|---|---|---|
| | Median | Range | Median | Range | Mann-Whitney (p) | p | r (95 % confidence interval) |
| 16S DNA (ng/µl) | 1.473 | 0.10-3.16 | 9.665 | 0.19-52.44 | 0.036 | | |
| IL-1β (pg/ml) | 670.7 | 104.8-3840 | 7668 | 29.73-59127 | 0.026 | 0.0001 | 0.78 (0.57-0.90) |
| IL-6 (pg/ml) | 430.8 | 62.6-20000 | 9.921 | 0.128-1381 | 0.0069 | 0.026 | -0.47 (-0.72 - -0.093) |

MFI = Median Fluorescent Intensity

| **Supplementary table 4. Baseline clinical characteristic comparing children to adults with PCD** | Controls (n=7) | PCD (n=27) | | |
|---|---|---|---|---|
| | | Children (n=10) | Adults (n=17) | Mann-Whitney (p) |
| Median age, years (range) | 46 (29-62) | 10.8 (7.6-17.7) | 29.6 (18.7-60.0) | |
| Female n (%) | 5 (71) | 5 (50) | 9 (53) | |
| FEV1pp median (range) | | 90 (75-116) | 60 (19-99) | 0.0004 |
| Presence of bronchiectasis n (%)[a] | | 6 (60) | 16 (94) | 0.047 |
| Genetically confirmed diagnosis *n* (%) | | 10 (100) | 11 (65) | 0.057 |
| Clinical diagnosis or unknown *n* (%) | | 0 (0) | 6 (35) | 0.057 |
| Ongoing exacerbation *n* (%) | | 1 (10) | 3 (18) | 0.99 |
| Positive sputum culture n (%) | | 6 (60) | 13 (87) | 0.18 |
| - *Haemophilus influenzae* n (%) | | 4 (40) | 8 (53) | |
| - *Pseudomonas aeruginosa* n (%) | | 0 (0) | 5 (33) | |
| - *Staphylococcus aureus* n (%) | | 2 (20) | 1 (7) | |
| Inhaled cortisone treatment n (%) | | 8 (80) | 7 (41) | 0.11 |
| Presence of antibiotic treatment n (%) | | 3 (30) | 7 (41) | 0.69 |

FEV1pp = Forced expiratory volume in one second in percent of predicted

[a] Diagnosed by chest CT (n=20) or chest tomosynthesis (n=7)

| **Supplementary table 5. Surface markers comparing macrophages stimulated with healthy sputum vs PCD sputum** | Healthy controls (n=7) | | PCD patients (n=27) | | |
|---|---|---|---|---|---|
| | Median | Range | Median | Range | Mann-Whitney (p) |
| CD163 (MFI) | 4155 | 2675-9049 | 11882 | 2388-104460 | 0.024 |
| CD16 (MFI) | 5901 | 2613-18251 | 23677 | 2596-52764 | 0.0032 |
| CD206 (MFI) | 19648 | 14519-42278 | 38153 | 11662-62815 | 0.044 |
| MerTK (MFI) | 43973 | 36812-54657 | 47664 | 19579-105296 | 0.43 |
| CD80 (MFI) | 8568 | 3844-12765 | 4672 | 2303-8777 | 0.035 |
| CD40 (MFI) | 23657 | 16268-40279 | 18138 | 12177-27425 | 0.013 |
| HLA-DR (MFI) | 78185 | 62066-241678 | 166092 | 42567-723030 | 0.11 |
| PD-L1 (MFI) | 8492 | 6399-13580 | 11462 | 4010-24652 | 0.12 |
| CD86 (MFI) | 521516 | 190659-873272 | 343892 | 102085-805254 | 0.50 |

MFI = Median Fluorescent Intensity

| **Supplementary table 6. Correlation of sputum cytokines to macrophage functions** | IL-1β (pg/ml) | | | IL-6(pg/ml) | | | 16S DNA | | |
|---|---|---|---|---|---|---|---|---|---|
| | r | 95 % confidence interval | p | r | 95 % confidence interval | p | r | 95 % confidence interval | p |
| Phagocytosis (MFI pHrodo) | 0.73 | 0.52 - 0.86 | <0.0001 | -0.32 | -0.60 - 0.033 | 0.067 | 0.47 | 0.14 - 0.70 | 0.0054 |
| IL-1β secretion (pg/ml) | -0.42 | -0.67 - -0.083 | 0.01 | 0.24 | -0.12 - 0.54 | 0.17 | -0.16 | -0.48 - 0.19 | 0.35 |
| IL-6 secretion (pg/ml) | -0.29 | -0.58 - 0.069 | 0.10 | 0.16 | -0.20 - 0.48 | 0.38 | -0.062 | -0.40 - 0.29 | 0.73 |
| IL-10 secretion (pg/ml) | 0.06 | -0.30 - 0.40 | 0.75 | -0.11 | -0.44 - 0.25 | 0.55 | 0.11 | -0.24 - 0.44 | 0.52 |

MFI = Median Fluorescent Intensity

| **Supplementary table 7. Effects of treatment regimens and age on macrophage polarization** | | CD163 (MFI) | CD16 (MFI) | CD206 (MFI) | CD80 (MFI) | CD40 (MFI) | Phagocytosis (MFI pHrodo) | IL-1β secretion (pg/ml) | IL-6 secretion (pg/ml) |
|---|---|---|---|---|---|---|---|---|---|
| No inhaled corticosteroids (n=8) | Median | 11616 | 24876 | 31044 | 4636 | 17344 | 203626 | 0.75 | 9.175 |
| | Range | 2898-36589 | 6016-44380 | 11662-44038 | 2303-8777 | 12177-23612 | 97752-300876 | 0.75-2.70 | 1.05-23.87 |
| Inhaled corticosteroids (=19) | Median | 12886 | 23290 | 46606 | 4672 | 18243 | 161361 | 0.75 | 12.02 |
| | Range | 2388-104460 | 2596-52764 | 23043-62815 | 2423-7681 | 13183-27425 | 42506-297341 | 0.75-8.271 | 1.68-101.3 |
| | Mann-Whitney (p) | 0.94 | 0.9 | 0.025 | 0.83 | 0.28 | 0.49 | 0.83 | 0.22 |
| No Azithromycin (n=21) | Median | 12886 | 22794 | 29265 | 4699 | 18138 | 177244 | 0.75 | 10.38 |
| | Range | 2388-104460 | 2596-52764 | 11662-62815 | 2303-8777 | 12177-23612 | 42506-300876 | 0.75-8.27 | 1.05-101.3 |
| Azithromycin (n=6) | Median | 10233 | 26175 | 50164 | 4638 | 18624 | 220168 | 0.75 | 14 |
| | Range | 3487-24638 | 3858-48296 | 16501-54828 | 2680-6198 | 14691-27425 | 79291-275671 | 0.75-2.33 | 2.11-60.67 |
| | Mann-Whitney (p) | 0.75 | 0.41 | 0.16 | 0.75 | 0.48 | 0.29 | 0.58 | 0.89 |
| Children (n=10) | Median | 12118 | 23431 | 44288 | 5761 | 18960 | 139466 | 2.369 | 30.5 |
| | Range | 2959-87319 | 4283-44380 | 16501-58483 | 2971-7175 | 15986-27425 | 42506-300876 | 0.75-8.271 | 4.779-101.3 |
| Adults (n=17) | Median | 11882 | 23677 | 36259 | 4573 | 17971 | 196837 | 0.75 | 5.504 |
| | Range | 2388-104460 | 2596-52764 | 11662-62815 | 2303-8777 | 12177-25340 | 79291-297341 | 0.75-2.579 | 1.053-60.67 |
| | Mann-Whitney (p) | 0.68 | 0.79 | 0.57 | 0.36 | 0.22 | 0.19 | 0.015 | 0.0004 |

MFI = Median Fluorescent Intensity
